# *Salmonella* Typhimurium in Iran: Contribution of molecular and IS*200* PCR methods in variants detection

**DOI:** 10.1371/journal.pone.0213726

**Published:** 2019-03-13

**Authors:** Reza Farahani Khaltabadi, Nader Shahrokhi, Mina Ebrahimi-Rad, Parastoo Ehsani

**Affiliations:** 1 Department of Molecular Biology, Pasteur Institute of Iran, Tehran, Iran; 2 Department of Molecular Biology, National Veterinary Reference Laboratory, Iranian Veterinary Organization, Tehran, Iran; 3 Department of Biochemistry, Pasteur Institute of Iran, Tehran, Iran; Laboratoire National de Santé, LUXEMBOURG

## Abstract

*Salmonella* Typhimurium, a zoonotic pathogen, is regarded as a major health and economic concern worldwide. Recently, monophasic variants of this serovar have been significantly associated with human gastroenteritis outbreaks globally, making its accurate identification essential for epidemiological and control purposes. We have identified and analyzed 150 *S*. Typhimurium from 884 *Salmonella* genus isolated from humans, domestic animals, poultry, food items and abattoirs origins. The *Salmonella* isolates were obtained from Iranian National Veterinary Reference Laboratories of 9 provinces during 2007–2016, and from five hospitals in Tehran in 2015. The isolates were evaluated biochemically, serologically, and by PCR amplification of *invA*, *mdh*, *STM4492*, *fliC*, *fljA*, *fljB*, *hin* genes, IS*200* and *DT104*. *invA* and *mdh* genes were used to confirm the *S*. Typhimurium serotype, *fliC* and *fljB* genes for determination of monophasic variants and amplification of IS*200* to discriminate the monophasic variants from the closely related serotypes. We identified 78.6% (118/150) as classical *S*. Typhimurium *(fliC*, *fljB* and IS*200* positive), 12.6% (19/150) were IS*200* negative from all isolates. DT104 is another marker for *S*.Typhimurium serovar typing. Contrary to EFSA guidelines 20.6% (19/29) of human isolates that lacked IS*200* insertion sequence, were confirmed as *S*.Typhimurium. Compared to the North American/European isolates the low prevalence of *fljB* negative 6% (9/150) and the high abundance of *fliC* negative 23.3% (35/150) isolates also were indicative of a different regional atypical population. Studies have shown that the prevalence of monophasic (*fljB*^*-*^*) S*. Typhimurium worldwide is promoted by the Swine industry. Thus, one reason for this high number of different atypical strains could be inhibition of swine breeding system (house hold and industry) in Iran. These results demonstrate a need for a modified identifying protocol to overcome the regional differences.

## Introduction

Non-typhoidal *Salmonellae* are a common cause of salmonellosis in humans and animals. Despite improvements in hygiene and food safety standards, the infection remains a common public health concern worldwide [1]. The spreading of infectious human *Salmonella* serovars occurs through consumption of contaminated food and water, contact with infected animals, international food trades and travelling by infected people within and among countries [2]. The burden of the infection is not limited to the associated morbidity and mortality costs of humans and animals, but it also includes the loss of trade and the consequent socioeconomic problems [2, 3]. *Salmonella*, *enterica* and *bongori*, are the two species of the genus with the former containing six subspecies *enterica*, *salamae*, *arizonae*, *diarizonae*, *houtenae and indica*. The most clinically important is *S*.*enterica* sub species *enterica* due to its common association with humans and warm-blooded animals and includes approximately 1500 serovars [4, 5]. Although potentially, all serovars of this subspecies could be pathogenic for humans, only about 80 of them account for nearly 99% of *Salmonella* infections in humans and domestic animals. *Salmonella* Typhimurium ranks among the top-five serovars, isolated from pigs, poultry, red meat and food-borne outbreaks in the European Union and the United States [4–8]. In Iran, the latest available data by the Center for Communicable Diseases and Control in 2011 showed that from 1038 reported food-borne diseases outbreaks, 5.1% were caused by *Salmonella* isolates, all serovars taken together [9].

Culture-based identification of *Salmonella* utilizes specific media and biochemical tests, followed by serotyping using special sera to determine the antigenic attributes of the isolated strains. This type of identification is based on the presence of somatic (O) and flagellar (H) antigens, according to the White-Kauffmann-Le minor scheme [10, 11]. The variable polysaccharide component of the cell wall specifies the O antigen of various serogroups and its presence, and variable expression of two flagellar antigens are determinants of the serovars [10, 11]. Alternative expression of *fliC* (H1) and *fljB* (H2) genes, controlled by *hin* locus, leads to phase-variation in biphasic motility in most of *Salmonella*s serovars [10–12]. *fljB* gene is located upstream of *fljA*, and FljBA mRNA codes both proteins. FljA protein represses the expression of phase I flagellar antigen, FliC [13]. The “phase variation” mechanism that controls both flagellar antigens in *Salmonella* spp., is an “on” and “off” switch, involving the reversible inversion of the DNA segment containing the fljB promoter and *hin* gene that codes the main DNA invertase regulator of phase variation. At “on” position, both FljA and FljB proteins are expressed, inhibiting *fliC* gene transcription, therefore, allowing the second flagellar phase to be expressed. Whereas, when the switch is at “off” position, neither genes are expressed, permitting *fliC* to be transcribed [14]. Determination of the antigenic formula of over 2600 serovars of *Salmonella* has been made possible by combining 46 different somatic and 114 different H antigens, identified in the White-Kauffmann-Le minor scheme [10–12, 15]. The antigenic formula for classic strains of *S*. Typhimuriumis1,4,[5],12:i:1,2 indicates the biphasic nature of the flagellar antigen, as specified by serotyping. However, variants lacking phase 1 (1,4,[5],12:-:1,2), phase 2 (1,4,[5],12:i:-) or both 1,4,[5],12:-:- are called “*Salmonella* Typhimurium-like” strains, as described by the European Food Safety Authority (EFSA). Except for the flagellar monophasic variant of 1,4,[5],12:i:- the other two variants have not been commonly the cause of salmonellosis in either humans or animals [10]. On the other hand, since 1990s, the monophasic serovar1,4,[5],12:i:-, has reportedly been associated with a significant number of human salmonellosis outbreaks in different countries, and has been isolated from domestic animals, poultry as well as food items [10, 15].

Serotyping by slide agglutination using polyclonal and monoclonal antisera for differentiation of a variety of organisms, has been a valuable tool for surveillance and epidemiological investigation of food-borne diseases [15]. However, this method is costly, time consuming and labor-intensive, while the procedure for detection of phase variation is technically demanding. Therefore, various DNA-based serotyping methods have been developed as alternative/complementary procedures to conventional sero-agglutination [10, 16]. Furthermore, the emergence and prevalence of *Salmonella* isolates lacking expression of phase 1 and/or phase 2 flagellar antigens have made the use of molecular methods essential [10]. PCR-based methods for identification and serotyping of *S*. Typhimurium are easy to perform, relatively inexpensive and reproducible [15, 17]. For serovar identification, amplification of *Salmonella*-specific *invA*, and for phase 1 and 2 antigens, *fliC*, *fljA*, *fljB*, *fliB-fliA* and *hin* genes have been proposed [18, 19]. Although, recently *STM 4492* coding for a putative cytoplasmic protein, and malic acid dehydrogenase (*mdh*) genes have been suggested as more suitable targets for Typhimurium serovar identification [20, 21].

*Salmonella* Typhimurium DT (definitive type) 104 carries multiple drug resistance genes on an integrated DNA segment in the genomic island 1. *Salmonella* Typhimurium receptor for phage type 104 has been detected since 1994 and reported worldwide due to increasing consumptions of related antibiotics. The receptors are flagella such as FliC and FljB on the bacterial surface [22] and identification of this phage is now one of the diagnostic tools for subtyping *S*. Typhimurium serovar and increasing antibiotic resistance trait [23].

To discriminate the *S*.Typhimurium variants from their closely-related serovars that share the same antigenic formula and phase one flagellar antigen, amplification of *fliB-fliA* intergenic region which carries a serovar specific copy of IS*200* insertion element and produces a 1000-bp amplicon instead of a 250-bp product is recommended by EFSA [10, 14, 19, 21, 23, 24]

Bacterial transposons are mobile genetic elements that promote DNA rearrangements and horizontal gene transfer of the host organism. Ellis *et al*. have shown that IS*200* encodes (707bp gene size) transposase mRNA (tnpA). The sRNA coded in mRNA, starts in 5'UTR of the gene, has a trans-acting effect repressing the expression of genes encoded within Salmonella Pathogenicity Island 1 (SPI-1) mRNA such as *invF*. However, despite its negative impact on the invasion of the bacteria into the non-phagocytic cells, it is recognized as a pathogenic marker [25].

In Iran, no published data are available on isolation of *S*.Typhimurium variant strains. Therefore, this study is aimed to investigate the variant strains among the isolates collected from humans, domestic animals, poultry, slaughter houses, red meat, chicken paste and non-domesticated birds, using sero-agglutination and PCR methods.

## Materials and methods

### Sample collection

The National Veterinary Reference Laboratories (NVRL), based in different provinces of Iran (S1 Fig), sent 819 *Salmonella* samples to the Iran Veterinary Organization from 2007–2016. The NVRL network is responsible for supervision and surveillance of farm animals’ health and diseases as well as the hygiene of the dependent industries in Iran. The samples had been identified as *Salmonella* at genus level through routine and random sampling using biochemical tests [26], and had been stored for further studies. Therefore, no live animals were used in our research. The origin of animals related samples were: feces from industrial animals [cattle, sheep, poultry (broiler, layer)]; fecal droppings from garden birds (sparrow, geese, duck, canary, parrot); the unprocessed food portions such as, red meat and chicken; the processed foods such as chicken paste; and water effluent from the slaughterhouses. There were no humans or human samples directly involved in this study. Sixty five *Salmonella* samples were isolated from outpatients in microbiology laboratories of five different hospitals during 2015. The isolated *Salmonella* samples were collected by Microbiological Laboratory of the hospital and handed to us with no information or codes regarding the patient's identity.

### Ethical approval

Although no human samples or live animals were involved in this study, this proposal has been reviewed and approved by the committee on the ethics of animal experiments of the Pasteur Institute of Iran (permit number: IR.PII.REC. 1394.83).

### Cultivation and seroagglutination

All *Salmonella* isolates were confirmed at the genus level using selective media, biochemical and molecular analysis [10, 19]. The isolates were cultivated overnight from transferred media into the Nutrient Broth, and subsequently subcultured on XLD selective media. A single colony from each plate was used for seroagglutination using polyclonal antibody against O antigen for B serogroup antigen (Mast, UK). The positive isolates were tested by polyclonal antibody against O4 antigen using purchased antisera (Mast, UK).

### Molecular identification and serotyping

A single colony from all the 884 isolates, irrespective of seroagglutination results, was sub-cultured in Luria Bertani broth (Merck, Germany) and DNA extracted using a PCR template extraction kit (11796828001 Roche, Germany), according to the manufacturer’s protocol. Purity and concentration of the extracted DNA was determined by spectrophotometer (NanodropX100, USA), and 10–20 ng was used as PCR templates.

Specific primer pairs for *invA*, *mdh*, *STM4492* and *DT104* genes were used for identification and subtyping and 150 *Salmonella* of the 884 isolates were confirmed as *S*.Typhimurium (Table 1). Seroagglutination of phase 1 antigen was performed on the 150 confirmed *Salmonella* isolates using anti H(i) antisera (Mast, UK) (Table 1). Phase 2 flagellar antigen was tested only for the *S*.Typhimurium isolated from humans and STM4492PCR-negative isolates using antisera from Mast, UK.

**Table 1:** Origin, number and distribution of *S*.Typhimurium isolates, identified in collected *Salmonella* from different provinces of Iran.

Sample Province	Human	Slaughter-house	Cattle	Sheep	Red meat	poultry	Chicken paste	Gees	Duck	Canary	Parrot	Sparrows	Total
Tehran	29	1	1	12	41	14	1	-	-	1	1	8	109
Qazvin	-	-	-	-	-	2	-	-	-	-	-	-	2
Qom	-	3	-	-	-	1	-	-	-	-	-	-	4
Mazandaran	-	-	-	-	-	6	-	1	1	-	-	-	8
Gilan	-	-	-	-	-	2	-	-	-	-	-	-	2
E. Azarbyjan	-	-	-	-	-	9	-	-	-	-	-	-	9
W. Azarbyjan	-	-	-	-	-	7	-	-	-	-	-	-	7
Sistan	-	-	5		1	2	-	-	-	-	-	-	8
Fars	-	-	-		-	1	-	-	-	-	-	-	1
Total	29	4	6	12	42	44	1	1	1	1	1	8	150

*fliC*, *fljA*, *fljB*, and *hin* genes, as well as *fliA–fliB* intergenic region were amplified to identify the *S*.Typhimurium serovar and its variants (Table 2). Amplifications were carried out in 20 μl using 2X master mix (Fermentas, Lithuania) with denaturation at 94°C for 5 min and 30 cycles of 94°C for 60 sec, annealing for each primer pair was as specified in the Table 2 for 30 sec and extension at 72°C for 1 min, followed by a final extension at 72°C for 10 min. Genomic DNA from *S*. Typhimurium (ATCC14028) and *S*. Enteritidis (ATCC13076) were used as positive and negative controls, respectively. Amplifications were repeated at least twice and each round of PCR contained a tube with no template DNA to monitor the potential contamination in the PCR components.

**Table 2:** List of primers used for serotyping of the samples.

Name of genes	Sequence (5’-3’)	Temp ^(°C)^	Amplicon (bp)	Ref.
*invA-F* *invA-R*	5’-aaacgttgaaaaactgagga 5’-tcgtcattccattacctacc	60	119	Saeki, 2013
*STM4492-F* *STM4492-R*	5’-acagcttggcctacgcgag 5’-agcaaccgttcggcctgac	62	759	Saeki, 2013
*Mdh-F* *Mdh-R*	5’-tgccaacggaagttgaagtg 5’-cgcattccaccacgcccttc	51	105	Bugarel, 2012
*fliC-F* *fliC-R*	5’-actcaggcttcccgtaacgc 5’-atagccatctttaccagttcc	50	550	Bugarel, 2012
*fljA-F* *fljA-R*	5’-tccgaagccagaatcaaattttcc 5’-tacgttttaatgatatccctgttcg	50	105	Bugarel, 2012
*fljB-F* *fljB-R*	5’-caacaacaacctgcagcgtgtgcg 5’-gccatatttcagcctctcgcccg	56	1389	Bugarel, 2012
*Hin-F* *Hin-R*	5’-cgccccggcctgaaacga 5’-cgactaatctgttcctgttcatgtt	50	334	Bugarel, 2012
*fliA-fliB-F* *fliA-fliB-R*	5’-ctggcgacgatctgtcgatg 5’-gcggtatacagtgaattcac	46	964	Bugarel, 2012
*DT104-F* *DT104-R*	5’-atgcgtttggtctcacagcc 5’-gctgaggccacggatattta	53	102	Alvarez, 2004

## Results

Out of 819 animals and 65 human Salmonellas isolates, 121 and 29 were positive for O4 (group B) antigen and PCR positive for *invA and mdh* genes respectively and were identified as *S*.Typhimurium (Table 1). The *STM4492*gene was not detected in 6.6% (10/150) of the strains. One of the *STM4492* negative strains belonged to human isolates (Table 3), three to red meat (Table 4), one to sheep (Table 5), four to chicken and one to chicken paste isolates (Table 6). Sero-agglutination tests for phase 1 and 2 flagellar antigens were repeated on these isolates, confirming the original identification and phase 2 serotyping was positive for one human, one red meat and one sheep sample.

**Table 3:** Slide-agglutinationof H(i) and H(1,2) flagellar antigens and PCR results of *Salmonella* isolates of human origin from five Tehran hospital.

Human Isolate	Year	Province	*invA*	*STM 4492*	*mdh*	Serotyp-ing (H:i)	*fliC*	*Serotyping (H*:*1 &2)*	*fljB*	*fljA*	*hin*	*fliA-B* IS*200 bp*	*Phage type DT104* PCR	*Hospital*
H8	2015	Tehran	+	+	+	-	-	+	+	-	+	1000	+	Milad
H9, H11, H12, H13	2015	Tehran	+	+	+	+	+	-	+	+	+	1000	+	Milad
H10, H14 H15, H16 H17, H20 H21, H22 H23, H25 H26, H27 H28	2015	Tehran	+	+	+	+	+	+	+	+	+	1000	+	Milad
H31	2015	Tehran	+	+	+	+	+	+	+	+	+	250	-	Emam
H32	2015	Tehran	+	+	+	+	+	+	+	+	+	250	-	Baghiyatallah
H18, H19	2015	Tehran	+	+	+	-	-	+	+	+	+	1000	-	Milad
H29	2015	Tehran	+	+	+	+	+	+	+	+	+	250	-	Milad
H33, H34	2015	Tehran	+	+	+	-	-	+	+	+	+	250	-	Baghiyatallah
H24	2015	Tehran	+	+	+	+	+	-	-	-	+	1000	+	Milad
H30	2015	Tehran	+	+	+	+	+	-	-	+	+	250	-	Milad
H96	2015	Tehran	+	-	+	+	+	+	+	+	+	1000	-	kodakan
H97	2015	Tehran	+	+	+	-	-	+	+	+	+	1000	+	Loghman

**Table 4:** Slide-agglutination of H(i) flagellar antigen and PCR results of *Salmonella* samples, isolated from red meat.

Red meat Isolate	Year	province	*invA*	*STM 4492*	*mdh*	*Serotyping—(H*:*i)*	*fliC*	*fljB*	*fljA*	*hin*	*fliA-B* IS*200*	*Phage type DT104 PCR*
R1	2013	Tehran	+	+	+	+	+	+	+	+	1000	-
R65	2011	Sistan	+	+	+	+	+	-	-	-	250	-
R73, R76, R78, R82, R84, R83, R88, R90, R94, R102, R120, R98, R99, R100, R101, R104, R105, R106, R119	2015	Tehran	+	+	+	+	+	+	+	+	1000	-
R77, R80	2015	Tehran	+	+	+	-	-	-	+	+	1000	-
R79, R138, R141, R142	2015	Tehran	+	+	+	+	+	+	+	+	1000	+
R81, R85, R86, R87, R89, R91, R92,R93	2015	Tehran	+	+	+	-	-	+	+	+	1000	-
R95	2015	Tehran	+	-	+	-	-	+	+	+	1000	-
R118	2015	Tehran	+	-	+	+	+	+	+	+	1000	-
R121	2015	Tehran	+	-	+	+	+	+	+	+	1000	+
R136, R137	2015	Tehran	+	+	+	-	-	+	+	+	1000	-
R139, R140	2015	Tehran	+	+	+	-	-	+	+	+	250	-

**Table 5:** Slide-agglutination of H(i) flagellar antigen and PCR results of *Salmonella* samples, isolated from cattle, sheep and slaughter houses.

Isolate	Source variety	Year	province	*invA*	*STM 4492*	*mdh*	*Serotyping—*(H:i)	*fliC*	*fljB*	*fljA*	*hin*	*fliA-B*IS*200*	*Phage type DT104*
Ca66	Cattle	2011	Sistan	+	+	+	+	+	+	+	+	250	+
Ca67, Ca68, Ca 69, Ca70	Cattle	2011	Sistan	+	+	+	-	-	+	+	+	250	+
Ca75	Cattle	2014	Tehran	+	+	+	-	-	+	+	+	1000	+
S124, S125, S127, S128, S129, S130, S131, S132, S133, S134, S135	Sheep	2015	Tehran	+	+	+	+	+	+	+	+	1000	+
S126	Sheep	2015	Tehran	+	-	+	+	+	+	+	+	1000	+
S-H7	Slaughter House	2012	Tehran	+	+	+	-	-	+	+	+	1000	+
S-H112, S-H113, S-H114	Slaughter House	2015	Qom	+	+	+	+	+	+	+	+	1000	-

**Table 6:** Slide-agglutination of H(i) flagellar antigen and PCR results of *Salmonella* isolated from poultry.

Isolate name	Source variety	Year	province	*invA*	*STM 4492*	*mdh*	*Serotyping- (H*:*i)*	*fliC*	*fljB*	*fljA*	*hin*	*fliA-B* IS*200*	*Phage type DT104*
C71	Chicken	2011	Sistan	+	+	+	-	-	-	+	+	1000	-
C2	Chicken	2013	Tehran	+	+	+	-	-	-	+	+	250	+
C3	Chicken	2013	Tehran	+	+	+	+	-	+	+	-	1000	-
C72	Chicken	2014	Tehran	+	+	+	-	-	-	+	+	1000	-
C103	Chicken	2015	Tehran	+	+	+	-	-	+	+	+	1000	+
C107	Chicken	2015	Tehran	+	+	+	-	-	+	+	+	250	+
C108	Chicken	2015	Tehran	+	+	+	-	-	+	+	+	1000	+
C109, C110	Chicken	2015	Tehran	+	-	+	-	-	+	+	+	1000	-
C111, C115, C116	Chicken	2015	Tehran	+	+	+	-	-	-	-	-	1000	+
C117	Chicken	2015	Tehran	+	+	+	+	+	-	-	-	250	+
C122	Chicken	2015	Tehran	+	-	+	+	+	+	+	+	1000	-
C123	Chicken	2015	Tehran	+	-	+	-	-	-	-	-	1000	-
C149, C150	Chicken	2016	W. Azarbyjan	+	+	+	+	+	-	+	+	1000	+
L35, L40	Layer	2008	Mazandaran	+	+	+	+	+	+	+	+	1000	-
L37	Layer	2008	Mazandaran	+	+	+	+	+	+	+	+	1000	+
L36, L38, L39	Layer	2008	Mazandaran	+	+	+	+	+	+	+	+	1000	-
L6	Layer	2011	Qom	+	+	+	-	-	+	+	+	1000	-
L4, L5	Layer	2012	Qazvin	+	+	+	-	-	+	+	+	1000	-
L62	Layer	2013	Gilan	+	+	+	-	-	+	+	+	1000	+
L63	Layer	2013	Gilan	+	+	+	+	+	-	+	+	1000	+
L74	Layer	2014	Fars	+	+	+	-	-	+	+	+	1000	-
B41, B42, B43, B44, B45, B46, B47	Broiler	2007	E. Azarbyjan	+	+	+	+	+	+	+	+	1000	-
B48	Broiler	2007	E. Azarbyjan	+	+	+	+	+	-	+	+	1000	-
B49	Broiler	2007	E. Azarbyjan	+	+	+	+	+	+	+	+	250	-
B64	Broiler	2011	Sistan	+	+	+	+	+	-	+	+	250	+
B144, B146, B147, B148	Broiler	2016	W. Azarbyjan	+	+	+	+	+	+	+	+	1000	+
B145	Broiler	2016	W. Azarbyjan	+	+	+	-	+	+	+	+	1000	+
Cp143	Chicken paste	2015	Tehran	+	-	+	-	-	+	+	+	1000	-

C: chicken, L: Laying chicken, B: Broiler, Cp: Chicken paste

Presence of flagellar genes was investigated with primers recommended by the EFSA [10],

Amplification and expression of phase one flagellar antigen was observed in 79.3% (23/29) *Salmonella* Typhimurium from patients (Table 3). Of the remaining 6 isolates, 4 contained phase twoflagellar gene (*fljB*) but serotyping results were negative and two were both genetically and phenotypically negative. The concomitant presence of each *fljB* and *fliC* genes and positive seroagglutination occurred in 86.2% (25/29) and 100% (29/29), respectively (Table 3).

Identification of DT104 subtype of *S*. Typhimurium showed that 43.3% (65/150) of *S*. Typhimurium isolates belonged to DT104 subtype. The results from five hospitals has shown that 95.0% (19/20) of positive DT104 isolates were collected from one hospital with three different flagellar genotype variants. The most prevalent clone of human patient isolates are diphasic isolates 58% (17/29) and the prevalence of negative phase one or two was similar in the remaining isolates(20.6% (6/29) Table 3).

The *fljB* gene was detected in 92.8% (39/42) of isolates from red meat and the PCR product for intergenic region (*fliA-fliB*) in all of these strains was the expected 1000 bp, except two isolates which produced a 250 bp amplicon and 11.9% (5/42) of the isolates from red meats sold in shops were of DT104subtype (Table 4). But 100% (18/18) of the *Salmonella*s strains from live cattle and sheep were of DT104 phage type (Table 5).

In Sistan province, 87.5% (7/8) of isolates in 2011 showed 250-bp amplicon for *fliA-fliB*, which is the highest, and had different genetic pattern for flagellar genes. Out of these isolates one was from red meat (Table 4), five from cattle (Table 5), and one from chicken (Table 6). Table 5 shows that 100% of the isolates from cattle, sheep and slaughter house carried *fljB* gene and 86.3% (19/22) were positive for DT104, which was the highest among the sample groups.

The most variation of *S*.Typhimurium isolates in genotype and in distribution belonged to the chicken group. The gene, *fljB*, was not found in 28.8% (13/45) of the isolates of poultry and chicken paste, but 23% (3/13) of these isolates showed 250bp amplification products for *fliA-fliB* from all provinces in different years. The remaining 71.1% (32/45) of the isolates were *fljB* positive and only two of them produced a 250-bp product for *fliA-fliB* region (Table 6).The majority of the genetically non motile *S*.Typhimurium[77.7% (7/9)] were found in the poultry group and the remaining two genetically non motile isolates belonged to the red meat (R77 and R80). The expression of phase one flagellar antigen was not observed in 42.2% (19/45) of the isolates; however, there is a discrepancy where one isolate(C3) gave positive agglutination results, even though the gene for ‘i’ antigen was not detected by PCR (Table 6). The presence of *DT104*gene cluster and the lack of IS*200* were detected in 42.2% (19/45) and 11.1% (5/45) of the isolates, respectively (Table 6).

Slide agglutination on 47 bacteria isolated from chicken samples with antisera for phase one showed negative result in 42.5% (20/47) of the isolates (Table 6) which was the highest among all the categories.

All 12 *Salmonella* samples in wild birds group, that were isolated in 2009 were positive for both phase 1 and 2 antigen genes by PCR and the phase 1 by slide agglutination. All the isolates had *fliA-fliB* region of 1000bp (Table 7)

**Table 7:** Slide-agglutination of H(i) flagellar antigen and PCR results of *Salmonella* samples, isolated from wild birds.

Isolate	Source variety	Year	province	*invA*	*STM 4492*	*mdh*	*Serotyping—(H*:*i)*	*fliC*	*fljB*	*fljA*	*hin*	*fliA-B* IS*200*	*Phage type DT104*
Sp50, Sp51, Sp52, Sp 54, Sp55, Sp56, Sp57	Sparrow	2009	Tehran	+	+	+	**+**	**+**	+	+	+	1000	-
Sp53	Sparrow	2009	Tehran	+	+	+	**+**	**+**	+	+	+	1000	+
G58	Gees	2009	Mazandran	+	+	+	**+**	**+**	+	+	+	1000	-
D59	Duck	2009	Mazandran	+	+	+	**+**	**+**	+	+	+	1000	-
Cy60	Canary	2009	Tehran	+	+	+	**+**	**+**	+	+	+	1000	-
P61	Parrot	2009	Tehran	+	+	+	**+**	**+**	**+**	**+**	**+**	1000	+

The results of testing different markers and genes produced 11 genetic groups of variants.

The most common isolated *Salmonella* variant (group I) was genetically positive for all the tested genes (Table 8). Followed by VII variants (23.3% (35/150) lacking *fliC* gene and protein) (Table 8).

**Table 8:** Different variants of Salmonella isolates regarding Slide-agglutination and PCR results.

Variants	Total count	Serology (H:i)	*liC*	*fljB*	*fljA*	*Hin*	IS*200*		*DT104*		flagellar phase prediction based on genotype		
							250 bp	+1000 bp	-	+	Non motile absence of both genes	Diphasic presence of both genes	Monophasic presence of either *flic* gene or *fljB*
I	93	+	+	+	+	+	4	89	51	42	0	93	0
II	6	+	+	-	+	+	2	4	2	4	6	0	0
III	1	+	+	-	-	+	0	1	0	1	1	0	0
IV	2	+	+	-	-	-	2	0	1	1	2	0	0
V	1	-	+	+	+	+	0	1	0	1	1	0	0
VI	1	+	-	+	+	-	0	1	1	0	1	0	0
VII	35	-	-	+	+	+	9	26	24	11	35	0	0
VIII	1	-	-	+	+	-	0	1	1	0	1	0	0
IX	1	-	-	+	-	+	0	1	0	1	1	0	0
X	5	-	-	-	+	+	1	4	4	1	0	0	5
XI	4	-	-	-	-	-	1	3	1	3	0	0	4
Sub total							19	131	85	65	48	93	9
Total	150						150		150		150		

Almost 62.6% (94/150) of *S*.Typhimurium were genotypically diphasic, 32% (48/150) were genotypically monophasic, and 6% (9/150) did not contain any flagellar genes. Classical *S*. Typhimurium accounted for 78.6% (119/150) of the isolates (*fljB and IS200* positive), and 8.0% (12/150) as EFSA monophasic variants (no *fljB* in II,III,IV,X,XI variants). Among all *S*.Typhimurium 25.3% (38/150) were genotypically monophasic for phase one antigen: no *fliC* (V,VII,VIII,IX). Out of 150 *S*. Typhimurium, 19 failed to produce IS*200* insertion sequence which are distributed in six variant groups of I,II,IV,VII,X,X1 (Table 8) of which, almost half belonged to the monophasic variant VII, 47% (9/19), lacking *fliC* gene and flagellar H(i) antigen.

## Discussion

Effective surveillance and control of pathogens require accurate identification and strain characterization. In this study, 150 out of 884 *Salmonella* isolates were identified as *S*.Typhimurium based on conventional and PCR methods, of which 121 were of non-human origin sent to the National Veterinary Laboratory and 29 from human diarrheal cases.

In this study *mdh* gene was amplified in all 150 isolates whereas, *STM4492* which had been suggested by Saeki *et al*. to be able to differentiate *S*.Typhimurium from 22 other *Salmonella* serovars failed to produce an amplicon in 6.6% of isolates [21].

All the isolates identified as *S*.Typhimurium, were tested for the presence of structural (*fliC* and *fljB)* and regulatory genes (*fljA* and *hin*) by PCR and phase one flagellar antigen (H(i) using seroagglutination [13].The results of serology and PCR for phase one antigen were the same except for 1.3% (2/150) of the isolates differing from Bugarel et al who reported a 12% (3/25) similarity between the results of the two tests (serology and gene absence for phase one) for S 1,4,[5],12:-:1,2 strains [19].

Based on the molecular results, 11 variants were identified. Similar to other studies Variant I was the most prevalent group. Although, monophasic 1,4,[5],12:i:- variant of *S*.Typhimurium has emerged as an important cause of salmonellosis, and it has been observed in many countries worldwide, this is the first report concerning their identification in Iran. As the prevalence of genotypically negative *fljB* was 12% (18/150) (variants II,III,IV, X,XI) of which 66.66% (12/18) were positive for IS*200* (Table 8), therefore, 8% (12/150) could be monophasic as recommended by EFSA.

In our study, following classical isolate, the monophasic serotypes of *fliC* was the second most prevalent group followed by *fljB*. The latter result is not in agreement with that reported by Bugarel *et al*. which showed that in France the genotypically negative *fljB* 28.9% (74/256) is the second most prevalent variant following the classical group [19].

Phase switching of flagellar protein expression is a common feature of *Salmonella* species [13]. In two isolates of variants III and IV that *fljA* gene is not detectable, the expression of *fliC* is in on position, while the *fljB* could still be subject to the phase variation (Table 8). For IV, VI and VIII variants that the *hin* gene is not present if the promoter for *fljB* gene is active, the expression of H(1,2) is locked in on or off position.

In this study, four out of 29 isolated*S*.Typhimurium1,4,[5],12:i:- were from hospital patients that carried *fljB* gene, similar to that reported by Bugarel *et al*. [19] as “inconsistent” variant. The reason for this could be mutation(s) in the *fljB* gene out of the primer-binding site affecting protein structure and consequently inhibiting the serological detection or deletion of promoter of *fljB* gene [27]. These isolates were genotypically considered as Typhimurium-like strains with some flaw in gene expression [19]. Similar events in *fliC* gene could explain the results obtained with the *fliC* positive, H:i negative isolate of group V. Moreover, mismatched nucleotides in primer annealing sites of *fliC* gene could have produced the *fliC* negative but H:i positive strain of the variant VI (Table 8).

Bugarel *et al*. have shown that 28.9% (74/256) and 1.1% (3/256) of the isolated strains from various origins(except humans) (Table 2) were genotypically *fljB* and *fliC* negative, respectively [19, 28]. Whereas, in our study using the same primers, the *fljB* and *fliC* negative isolates from various origins were 10.6% (16/150) and 27.3% (41/150), respectively. This result could represent a new pattern for prevalence of *S*.Typhimurium variants in Iran.

One of the parameters of a serotype spread in a community is the animal industry, studies have shown that the first isolates of monophasic *fljB* negatives were from poultry (1986) and later detected in pig industry (2003) [15]. Moreover, different studies have shown that the majority of the monophasic *Salmonella* strains (1,4,[5],12:i:-) were isolated from pigs and pork industry in Spain(1999) [28], beef and pork products in China(2015) [29] and pigs, and turkey in Great Britain[30]. In Iran, the meat industry is mostly based on poultry producing 2.2 million tons yearly being the seventh largest producer of chicken meat in the world [31]. On the other hand, pig is not bred in house-hold or in industry and there is no record of importing the pork products. This condition could eliminate the propagation and circulation of the well adapted monophasic *S*.1,4,[5],12:i:- variants of pork origin in Iran. To our knowledge there is no publication on prevalence of monophasic variants from other Islamic countries for comparison.

The profile of the genetic markers used for identification of monophasic *S*. Typhimurium have also been considered as indicators of the clonal lineage of these isolates [13, 19, 25, 32]. The “Spanish” and the “USA clone” of monophasic *S*.Typhimurium have been reported negative for *fljA* as well as *fljB*, which are a genotypic combination observed in only one monophasic *S*.Typhimurium (1,4,[5],12:i:-) isolated from human (Table 3) and in 2/7 genotypically monophasic *fljB* negative animal isolates (Tables 4 and 6(. Therefore, most of the Iranian monophasic *fljB* negative isolates lacked only the *fljB* gene (6/9) (Table 8) and were not similar to Spanish/ French (*fljA*^-^, *fljB*^-^, *hin*^-^) and USA (*flj*A^-^, *fljB*^-^) clones [19].

In this study, 6% (9/150) of the isolates (X, XI) were genotypically non-motile. Two were from red meat and seven from chicken. These isolates were mostly from Tehran (8/9) that were collected in 2015. In the study by Bugarel *et al*., the rate of non-motile was close to 6.6% (17/256), but none of the isolates were genotypically negative for both genes [19].

Some of the factors that affect the severity and prognosis of the disease induced by *S*.Typhimurium are the presence of DT104 prophage [33], flagellar antigens and IS*200* [25]. *S*.Typhimurium DT104 is a human food-borne and animal pathogen that carries DT104 phage. In our study, 150 samples were tested for DT104, the highest isolation rate was observed in samples from cattles (86.3% (19/22) Table 5), highlighting the importance of these in dessimination of DT104/U302 subtypes with possible phage-associated resistance genes. A review in 1995 from UK has shown that the prevalence of *S*.Typhimurium DT104 in cattle/sheep, swine and poultry was 70%, 36%, and 52%, respectively[34]. However, in our study, the prevalence of DT104 for cattle/sheep, human and chicken were 86.3%, 68.8%, and 42.2%, respectively. Almost half the *S*. Typhimurium isolates (45.1% (42/93) in group I or in classic group belonged to this subtype (Table 8).

Flagella play an important role as receptor for DT104 [22] and among the 65 confirmed DT104 positive isolates 64.6% (42/65) carried both flagellar genes; 18.6% (12/65) were positive for *fljB* and negative for *fliC* gene and 9.2% (6/65) were *fliC* positive and *fljB* negative (Table 8). This is in accordance with Shin *et al*. that showed the presence of both flagella is important as receptor for DT104 phage that belongs to the group FI and FII of phage families [22].

One of the important markers of *S*. Typhimurium is IS*200* sequence located in *fliA-B* region, downstream of *fliC* gene [35]. According to the EFSA guidelines for *S*. Typhimurium identification, both classical and *fljB*^*-*^ monophasic variants carry IS*200* in *fliB-fliA* intergenic region by which *S*.Typhimurium and its variant are distinguished from other *Salmonella* serovars namely: *S*. Lagos, *S*. Agama, *S*. Farsta, *S*. Tsevie, *S*. Gloucester, and *S*. Tumodi [10]. Here in human category (Table 4), 3/13 classical and 1/6 monophasicS.1,4,[5],12:i:- of confirmed *S*.Typhimurium serovar showed deletion of IS*200*. Moreover, 12.7% (19/150) of all isolated *S*. Typhimurium in this study (Table 8) lacked IS*200* and thus did not support the EFSA suggestion that IS*200* should be present in all variants of *S*.Typhimurium. Bugarel *et al* has also shown that IS*200* is absent in 0.4% (1/256) of the European isolates [19]

Beuzone *et al*. have shown that IS*200* could rearrange chromosome up to several kb near its insertion site when it transposes [35]. The proximity (<1.5 kb) of the *fliC* gene and IS*200* insertion site, may also affect the presence of *fliC* gene when IS*200* transposes. In our study, the comparison of the ratio of *S*.Typhimurium lacking IS*200* among variant I (4.3%) and variant VII (25.7%) showed 5.97 times increase in the absence of IS*200* in variant VII that lacks *fliC* gene (Table 8).

## Conclusion

Our results showed that *S*. Typhimurium with IS*200*deletion is a fairly common serotype among the Iranian *Salmonella* isolates of human and non-human origins. Therefore, with the procedure recommended by EFSA some of the variants will not be correctly identified and therefore missed. In addition, with 34.3% prevalence of DT104 subtype that could carry resistant genes, among the atypical strains highlight the importance of adaptation of new guidelines for identification of all the variants.

## Supporting information

S1 Fig Location of provinces that *Salmonella* samples were obtained. (TIF)

S1 File The raw data of S*almonella* samples. Molecular detection of genes involved in the study.
(XLSX)
