# Supplementary figures and images for: Salmonella Typhimurium in Iran: Contribution of molecular and IS200 PCR methods in variants detection
Source: PLoS One. 2019 Mar 13;14(3):e0213726. doi: 10.1371/journal.pone.0213726 (PMC6415898; doi:10.1371/journal.pone.0213726)

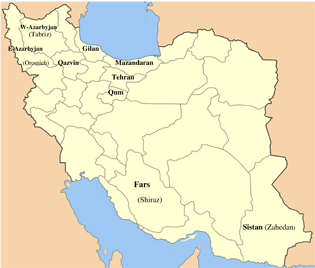

Supplement: S1 Fig — (TIF) [file pone.0213726.s001.tif]
